# Risk of Gastrointestinal Bleeding Among Dabigatran Users – A Self Controlled Case Series Analysis

**DOI:** 10.1038/srep40120

**Published:** 2017-01-20

**Authors:** Wenze Tang, Hsien-Yen Chang, Meijia Zhou, Sonal Singh

**Affiliations:** 1 Department of Epidemiology and Biostatistics, Milken Institute School of Public Health, The George Washington University, Washington, District of Columbia, USA; 2 Center for Drug Safety and Effectiveness, Johns Hopkins Bloomberg School of Public Health, Baltimore, Maryland, USA; 3 Department of Health Policy & Management, Bloomberg School of Public Health, Baltimore, USA; 4 Division of General Internal Medicine, Johns Hopkins Medicine, Baltimore, Maryland, USA; 5 Department of Family Medicine and Community Health, University of Massachusetts Medical School, Worcester, Massachusetts, USA

## Abstract

This article aims to evaluate the real world risk of gastrointestinal bleeding among users naïve to dabigatran. We adopted a self-controlled case series design. We sampled 1215 eligible adult participants who were continuous insured users between July 1, 2010 and March 31, 2012 with use of dabigatran and at least one gastrointestinal bleeding episode. We used a conditional Poisson regression to estimate incidence rate ratios. The population consisted of 64.69% of male and 60.25% patients equal to or greater than age 65 at start of observation. After adjustment for time-variant confounders, the incidence rate of gastrointestinal bleeding was similar during dabigatran risk period and non-exposed period (incidence rate ratio [IRR] = 1.01, 95% confidence interval [CI] 0.90, 1.15). There was no significant difference in GI incidence rate between periods of dabigatran and warfarin (IRR = 0.99, 95% CI 0.75–1.31). Among this database of young and healthy participants, dabigatran was not associated with increased incidence rate of GI bleeding compared with non-exposed period among naïve dabigatran users. We did not detect an increased risk of GI bleeding over dabigatran vs warfarin risk period. Along with other studies on safety and effectiveness, this study should help clinicians choose the appropriate anticoagulant for their patients.

Oral anticoagulants are effective in preventing thromboembolic events among patients with atrial fibrillation^1^. In 2010, dabigatran was approved by the United States Food and Drug Administration as an alternative to warfarin, a widely used anticoagulant. Dabigatran etexilate, a prodrug, is converted into its active moiety, dabigatran, in the gastrointestinal (GI) tract, plasma and liver^2^. Its relative advantages include fewer medication interactions, no required monitoring and simple dosing. Dabigatran has transient pharmacologic effects; the half-life of dabigatran is 10–12 hours only^3^. It is excreted primarily via kidney, thus patients with impaired renal function have a prolonged half-life^4^. The relative efficacy and non-inferiority of dabigatran compared to warfarin have been established by clinical trials^5^,^6^. In Randomized Evaluation of Long-Term Anticoagulant Therapy (RE-LY) trial, dabigatran administered at a dose of 150 mg twice per day was associated with lower rates of stroke, systemic embolism and serious intracranial bleeding compared to warfarin; dabigatran is also associated with higher rates of GI bleeding (relative risk 1.50, 95% confidence interval [CI]: 1.19–1.89)^7^,^8^, especially among older patients and patients with obesity and impaired renal function^9^. However, the real world effectiveness and safety of dabigatran also need to be evaluated in observational studies, as clinical trials were conducted on selective patients^10^. Several observational studies have adopted a traditional cohort design to evaluate this association^11^,^12^,^13^,^14^.

Despite the various potential adverse effects of anticoagulant, GI bleeding is of particular concern due to its mortality and morbidity. In the United States, more than 140,000 hospital admissions involved the principal diagnoses of GI hemorrhage not otherwise specified, causing in total 612,000 days of hospital stay and an aggregate cost of over 1 trillion USD in 2009^15^; among these hospitalized, 3.5% died. In the United Kingdom (UK), 103 out of 100,000 adults experience upper GI bleeding per year; the rate is even higher among the elderly^16^. Those hospitalized in UK for acute upper GI bleeding had a mortality rate of 10% in 2007^17^. Among patients with atrial fibrillation in the UK, fatality rates of GI bleeding attributable to warfarin use is as high as 6%^18^.

We used a self-controlled case series design to compare the relative safety of dabigatran regarding the risk of GI bleeding among users naïve to dabigatran using claims data. Claims data has been used widely in health services research and pharmacoepidemiology studies to examine the real-world impact, relative to the restricted environment under randomized controlled studies^12^,^15^,^19^,^20^. The self-controlled case series (SCCS) design has been used to study association between transient exposure and acute outcome of interest^21^,^22^. SCCS examines how incidence rate of outcome within the same individual differs between the periods exposed to drugs under investigation and the non-exposed period (Fig. 1). The design has four implications: first, each subject can have multiple exposures and experience multiple events; second, the risk period with drug exposure can be compared against another drug exposure or the non-exposed period; third, the observation period can also serve as a period to determine study population eligibility (e.g. the study population is restricted to individuals who experienced at least one outcome event); fourth, the intra-person comparison implicitly controls for all time-invariant confounders^23^.

## Methods

### Data Source

We used commercial LifeLink Health Plan Claims Data compiled by IMS Health under a data usage agreement. Data files can be made available to academic researchers on request as long as it does not violate the terms of agreement and provided that the researchers sign a confidentiality statement and receive IMS approval for their request. This dataset contained private health insurance plan information from managed care plans and other sources (such as Medicare and Medicaid) across the United States. It is considered to be representative of the nation’s commercially insured population^24^. Two parts of the dataset were used; enrollment files contained each individual’s enrollment history (both medical and pharmacy), region, together with individual demographics. Claims files included all the diagnosis documented as International Classification Disease, Ninth Revision, Clinical Modification (ICD-9), procedures as the Current Procedural Terminology codes (CPT-4) or the Healthcare Procedural Coding System (HCPCS), prescription as National Drug Code (NDC), date of services, and the number of days with drug supply. For age information, only year of birth was available. Therefore, the birth date of each individual was set to the middle day of the year (i.e. June 30). The data source included de-identified information so this project was deemed not to be human subject research by the institutional review board of the Johns Hopkins Bloomberg School of Public Health.

### Inclusion Criteria

Our observation period spanned from July 1, 2010 to March 31, 2012 (i.e. 21 months). We restricted our study subjects to those (1) not having exposed to warfarin or dabigatran between July 1,2010 and October 19, 2010, when dabigatran first obtained administrative approval (i.e. naive user design), (2) never having exposed to rivaroxaban throughout the observation period, (3) having continuous medical and pharmacy enrollment throughout the observation period, (4) at least 18 years old on July 1, 2010, (5) having at least one unique GI bleeding case during the observation period, and (6) having experienced dabigatran risk period and at least one of the two following periods: period with no exposure (i.e. non-exposed period) and warfarin risk period; participants with only warfarin, dabigatran or non-exposed risk period were removed as these participants did not transition between periods of exposure/non-exposed and therefore did not contribute to SCCS analysis.

### Outcome of Interest: GI Bleeding

We used ICD-9 and CPT codes to identify 7 types of GI bleeding cases (Appendix 2). This algorithm has been validated in a previous study and the positive predictive value was 83%^25^. We only counted the first GI bleeding case if two cases of the same type occurred within 7 days based on the acute course of most episodes of GI bleeding.

### Sample Size

We assumed that the episodes of GI bleeding were independent. We estimated that 398 episodes of GI bleeding would be needed during dabigatran risk periods to detect an effect size of 1.25 comparing dabigatran against warfarin risk period with 80% power and 95% confidence interval^26^.

### Exposure/Non-exposed Period Definition

The exposed risk period of dabigatran and warfarin were separately defined as the days of supply following the dispense event plus a grace period of 14 days^3^; the grace period is the washout period following the end of medication supply to account for residual effects of drug exposure.

**Figure 1:**
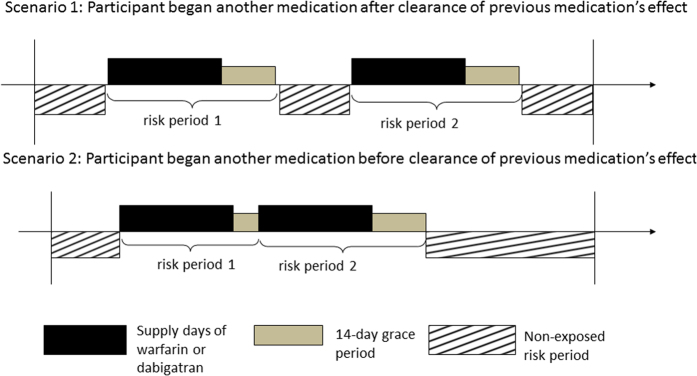
Diagram of typical risk periods in self-controlled case series design.

Warfarin exposure was considered continuous if two warfarin risk periods overlapped. The same was applied to dabigatran. If the risk periods of one warfarin and another dabigatran overlapped (e.g. the patient switched from warfarin to dabigatran or vice versa), the start date of second drug’s risk period was considered the end date of the first drug’s risk period. The unassigned time period where participant took neither warfarin nor dabigatran was designated as the non-exposed period (Fig. 1).

### Statistical Analysis

We used conditional Poisson regression model to estimate the incidence rate ratio (IRR). We used SAS version 9.3 for data management and analyses. SAS coding for the data management and analyses is readily accessible as a supplementary file.

We also adjusted for time-dependent covariates to minimize the impact of confounders^26^. We considered the following variables as potential time-dependent confounders: age groups, co-medication use [proton pump inhibitors (PPIs), steroids, P-glycoprotein (PGP) inhibitors and nonsteroidal anti-inflammatory drugs (NSAIDs)], development of specific chronic conditions (renal failure, trauma and H. pylori infection) and the HAS-BLED bleeding score to concomitantly account for bleeding-related risk factors including Hypertension, Abnormal renal/ liver function, Stroke, Bleeding history or predisposition, Labile international normalized ratio, Elderly and Drugs/alcohol^27^,^28^,^29^. The lower the HAS-BLED bleeding score, the less bleeding-related comorbidities presented. All time-variant covariates were treated as categorical. However, age group, development of renal failure and H pylori infection were excluded from adjusted model because less than 5% of the subjects experienced status change throughout the entire observational period in the primary analyses or any of the sensitivity analyses (Appendix 3). We divided the entire study period into seven 3-month segments and for each participant we generated a HAS-BLED score over each three-month period using ICD-9 diagnosis codes. We also designated an indicator of covariates for each of these periods; if a comedication was used for more than 14 days, the medication was considered to have been used during that three-month period. If certain chronic condition were recorded within one three-month period, that three-month period was considered exposed to that condition. These three-month periods containing HAS-BLED score as well as covariate indicators were merged with exposure risk periods. This way, all three-month periods could be further segmented by the risk periods of dabigatran, warfarin and non-exposure. We further categorized HAS-BLED scores into 3 levels (i.e. 0, 1, >=2) based on its distribution in adjusted model.

### Sensitivity Analyses

We conducted various sensitivity analyses to test the robustness of our results. We stratified risk periods to those with HAS-BLED bleeding score >= 1 and those with HAS-BLED bleeding score = 0 to test the impact of levels of bleeding risk on the outcome. We stratified risk periods into the above two levels to avoid a decrease in sample size. We also stratified subjects by age groups at baseline: those older than 65 versus 65 or younger.

To test the validity of exposure grace periods, we also used 7 days of grace period for both exposures. In addition, due to the inability of prescription data on warfarin to reliably estimate the days of warfarin exposure, 30 days of grace period at the end of warfarin prescription date were tested in the sensitivity analysis.

We also conducted an analysis using prevalent user design by allowing the inclusion of participants who had been using warfarin in the three months before dabigatran became available.

## Results

We identified 1,215 adult continuous enrollees with at least one GI bleeding event and dabigatran use between July 1, 2010 and March 31, 2012. Among these dabigatran users, 24.12% (i.e. 293 patients) switched between dabigatran and warfarin and 64.69% were male. At the onset of the study, 60.25% were older than 65 years. Nearly 70% of the patients were at the lowest two levels of bleeding risk score (i.e. 0 and 1) over the first 3 months of observation. Over the same period, 24.94%, 8.56%, 6.01% and 14.81% had used PPI, steroids, PGP inhibitors and NSAID respectively. Throughout the 21 months of the study, 14.24%, 40.58% and 1.81% of subjects developed renal failure, trauma and H. pylori infection, respectively (Table 1).

List of potential indications for dabigatran use were summarized in appendix 1 stratified by age groups, identified using ICD-9 code of atrial fibrillation and venous thromboembolism that took place during the 12 months prior to patient’s dabigatran initiation.

The mean exposed duration of dabigatran and warfarin use were 159 and 114 days respectively. The mean non-exposed duration was 448 days. Over the risk period of dabigatran, warfarin and non-exposed, there were 470, 84 and 1,159 episodes of GI bleeding during the risk period of dabigatran, warfarin and non-exposed risk periods, respectively (Table 2).

### Dabigatran vs Non-exposed Period

Compared with non-exposed period, the incidence rate of GI bleeding was 1.23 times higher for patients during the dabigatran risk period (IRR: 1.23, 95% CI: 1.09–1.39). After adjusting for bleeding score, use of NSAIDs, PPIs, PGP inhibitors and steroid medications as well as development of trauma, dabigatran showed similar risk of GI bleeding with non-exposed period (IRR: 1.01, 95% CI: 0.90–1.15) (Table 3).

### Dabigatran vs Warfarin Period

In both unadjusted and adjusted model, compared with warfarin risk period, dabigatran risk period was not associated with significant increase in the risk of GI bleeding (unadjusted IRR: 1.02, 95% CI: 0.77–1.34, adjusted IRR: 0.99, 95% CI: 0.75–1.31) (Table 3).

Among all available confounders, HAS-BLED score equal to or greater than 1, and concomitant use of PPIs were significant risk factors with p < 0.05 (Appendix 4).

### Sensitivity Analyses

In stratified analyses, IRRs comparing dabigatran risk period against non-exposed period were 1.05 (95% CI: 0.92–1.20) and 1.03 (95% CI: 0.71–1.51) for risk periods with HAS-BLED score > 0 and HAS-BLED score = 0 respectively. IRRs comparing dabigatran risk period against warfarin risk period were 1.00 (95%CI: 0.74–1.33) and 0.65 (95% CI: 0.25, 1.71), for risk periods with HAS-BLED score > 0 and HAS-BLED score = 0 respectively (Appendix 5 and Appendix 6).

The cohort younger than or equal to 65 years old had a significantly increased risk during warfarin risk periods compared to non-exposed period (IRR = 2.73, 95%CI: 1.14–6.53) while IRR comparing dabigatran vs non-exposed risk periods were similar in both younger and older cohort (Age > 65: IRR = 1.02, 95CI: 0.90–1.15 and age <= 65 years: IRR = 0.98, 95CI: 0.58–1.65). There was a 64% significant decrease in incidence rate among the younger cohort when they were on dabigatran compared to when they were on warfarin (IRR = 0.36, 95% CI: 0.14–0.93, Appendix 7).

Sensitivity analyses with various grace period assumptions produced similar results to the primary analyses (Appendix 4).

By including prevalent warfarin users into our study population (who switched to dabigatran after dabigatran became available), we found that after adjusting for time-dependent covariates, dabigatran risk period was associated with 12% significantly higher risk of GI bleeding compared with the non-exposed period (95% CI: 1.02–1.23). IRR comparing dabigatran risk period against warfarin risk period also indicated significantly increased risk of GI bleeding (IRR = 1.13, 95CI: 1.00–1.27) (Appendix 8).

## Discussion

In this group of the commercially insured enrollees with no prior use of warfarin, periods of dabigatran use was not associated with increased risk of GI bleeding, compared to periods of non-exposure. Dabigatran and warfarin use seem to be associated with similar risk of GI bleeding.

Our findings of similar risk of GI bleeding associated with dabigatran and warfarin bears similarities and differences with results of recent randomized controlled trials and some observational studies. One previous meta-analyses of clinical trials reported an significantly increased risk for GI bleeding associated with dabigatran compared to warfarin (OR = 1.58, 95% CI: 1.29–1.93)^9^. An FDA study focusing on the elderly Medicare patients with non-valvular atrial fibrillation also found a similarly increased risk with dabigatran relative to warfarin (OR = 1.28, 95% CI: 1.14–1.44)^13^. However, our result is in line with most recent retrospective cohort studies from Denmark and US both of which reported no significant increase in GI bleeding with dabigatran relative to warfarin^11^,^12^,^14^. The Danish study found that naïve dabigatran users on a 110 mg regimen had 47% significantly lower risk of GI bleeding compared with warfarin users(IRR = 0.53, 95%CI: 0.28–0.98)) while dabigatran users who switched from warfarin had insignificant higher risk of GI bleeding compared with persistent warfarin users(IRR = 1.22, 95% CI: 0.73–2.03)^14^. Our analysis of prevalent warfarin users suggests that switchers from warfarin to dabigatran are at significantly higher risk of developing GI bleeding, relative to warfarin exposure or non-exposed.

Our statistically insignificant results when comparing new dabigatran use to warfarin could be explained by the younger cohort and its associated lower comorbidity level, as indicated by our stratified analyses. The relatively short average duration of dabigatran and warfarin might limit the accumulation of bleeding risk. In contrast to our study, dabigatran was found to significantly increase the risk of GI bleeding in the Medicare cohort compared to warfarin, as it involved older participants with comorbidities^30^.

Our study provides several novel findings on the gastrointestinal safety of dabigatran topic. First, we were able to assess the relative risk of GI bleeding comparing dabigatran use against no anticoagulant use whereas previous studies have only been able to provide estimates comparing dabigatran use against warfarin. Second, unlike a case-control study with a binary outcome or cohort study with a time-to-event/censoring, the SCCS design allows for recurrent episodes of GI bleeding and estimates the average risk over a duration of person-time (incidence rate)^31^. In addition, unlike other studies where confounders may only be treated as baseline constant, we controlled for important confounders including overall bleeding-related risk factors that are time-variant. This allowed for within-person change of risk level along time. Our study showed that higher bleeding-related risk level of the patients and PPI use as co-medication were both strong and consistent confounders for the association between dabigatran and GI bleeding. We used a validated algorithm to identify GI bleeding cases and performed several sensitivity analyses to evaluate the robustness of our results.

Our study has some limitations. First, the assumption that previous GI bleeding would not alter the probability of being exposed to the drugs following that event could be violated and introduce reverse causality bias; in clinical practice, patient are usually asked to temporarily suspend novel anticoagulant for 2–3 weeks after a GI bleeding event occurs. In order to adjust for the reverse causality bias, we used the standard bidirectional design setting, where risk periods before(i.e. pre-exposure risk periods) and after the first exposure event were both included for analysis^21^,^32^. Although the exact timing of drug discontinuation is unknown and will depend on clinical context, it is less likely that the anticoagulant disruption after a GI bleeding event would either last longer than 3 months or lead to more immediate re-exposure to anticoagulant as the clinical conditions which mandated anticoagulation (e.g. atrial fibrillation and venous thromboembolism) pose a continuing risk for patients. As a result, our estimate could be biased towards either direction but is more likely to be biased away from the null if reverse causality persists. Readers should interpret our results with such acknowledgment. Second, the drug dispensing and supply information in data may not necessarily represent real world adherence. Although we used 7, 14 and 30 days of grace period for warfarin, risk window definition of warfarin was still somewhat uncertain given the inability of prescription data on warfarin to accurately estimate its duration. PPI and NSAID use might also be underestimated as both are available over the counter and we did not have information on aspirin use. Third, even though we used a validated algorithm to identify GI bleeding, potential misclassification of outcome is possible since we did not validate the claims in this database. This would bias our results to the null. Fourth, our findings may be subject to unmeasured time-variant confounding, such as laboratory testing of prothrombin times, and other time-variant residual confounding. Fifth, we did not assess long-term outcomes such as stroke due to the fact that a much longer follow-up time is necessary for this study. Sixth, the administrative claims we used did not have sufficient information on mortality, therefore we did not exclude participants who died immediately after the last outcome event but before the end of observation period. This could bias the estimate towards either direction. Seventh, HAS-BLED score has only been validated for warfarin’s GI bleeding risk among atrial fibrillation patients, therefore, it may not be generalizable to other populations. Finally, our non-significant estimates of GI bleeding associated with warfarin exposed period vs non-exposed risk periods should be interpreted with caution because of insufficient sample size.

## Conclusion

In this database of relatively young and healthy commercially insured participants, dabigatran was not associated with increased incidence rate of GI bleeding compared with non-exposed period among new dabigatran users. We did not detect an increased risk of GI bleeding over dabigatran risk period vs warfarin risk period. Our study suggested age cohort, time-variant bleeding-related comorbidities and whether a subject is naïve dabigatran user could significantly modify their risk of GI bleeding associated with dabigatran and warfarin. Our study should provide additional information on the relative safety of dabigatran. Along with other studies on the efficacy, effectiveness and safety of anticoagulants, this study would help clinicians prescribe the appropriate anticoagulant for their patients.

## Additional Information

**How to cite this article**: Tang, W. *et al*. Risk of Gastrointestinal Bleeding Among Dabigatran Users – A Self Controlled Case Series Analysis. *Sci. Rep.* **7**, 40120; doi: 10.1038/srep40120 (2017).

**Publisher's note:** Springer Nature remains neutral with regard to jurisdictional claims in published maps and institutional affiliations.

## Supplementary Material

Supplementary Information

## Figures and Tables

**Table 1:** Characteristics of dabigatran users during the study period (N = 1215).

Variables	N	%
Age Groups^a^		
18–44	23	1.89
45–54	103	8.48
55–64	357	29.38
65+	732	60.25
Sex		
Male	786	64.69
Region		
East	110	9.05
North West	339	27.90
South	606	49.88
West	160	13.17
HAS-BLED Score Level over first three months^b^		
0	308	25.35
1	541	44.53
>=2	366	30.12
Co-medication over first three months^b^		
Proton pump inhibitors	303	24.94
Steroid	104	8.56
P-glycoprotein Inhibitor	73	6.01
Nonsteroidal anti-inflammatory drugs	180	14.81
Ever developed listed chronic conditions^c^		
renal failure	173	14.24
trauma	493	40.58
H. pylori infection	22	1.81

^a^Summarized based on the information on Jul 01, 2010.

^b^Summarized over the first three months of the observation period, i.e. from Jul 01, 2010 to Sep 30, 2010.

^c^Summarized based on the whole observation period.

**Table 2:** Number of cases of GI bleeding within each exposure risk period (Number of gastrointestinal bleeding = 1,713).

Variables	No Drug Exposure	Warfarin exposure	Dabigatran exposure
#of patients with the specified risk period	1215	293	1215
#of GI bleeding episodes unique to each of the risk period	1159	84	470
Mean individual risk period in days (Range)	448(101–632)	114(1–431)	159 (1–503)
Median individual risk period in days (IQR)	476 (356–555)	89 (43–153)	128 (50–241)

GI-Gastrointestinal, IQR-Interquartile range.

**Table 3:** Incidence rate ratio of gastrointestinal bleeding by different groups.

Drug Comparison	IRR (95% CI)	
	Unadjusted model	Adjusted model
Dabigatran vs Non-exposed	1.23* (1.09, 1.39)	1.01 (0.90, 1.15)
Warfarin vs Non-exposed	1.21 (0.93, 1.57)	1.02 (0.78, 1.33)
Dabigatran vs Warfarin	1.02 (0.77, 1.34)	0.99 (0.75, 1.31)

IRR-Incidence rate ratio, CI-Confidence interval.

*p < 0.05.
