# Supplementary material for: Risk of Gastrointestinal Bleeding Among Dabigatran Users – A Self Controlled Case Series Analysis
Source: Sci Rep. 2017 Jan 20;7:40120. doi: 10.1038/srep40120 (PMC5247708; doi:10.1038/srep40120)
Supplement: Supplementary Information [file srep40120-s1.pdf]

RISK OF GASTROINTESTINAL BLEEDING AMONG DABIGATRAN USERS – A SELF CONTROLLED CASE SERIES ANALYSIS

Wenze Tang[1], MPH
Hsien-Yen Chang[23], PhD
Meijia Zhou[2], MHS
Sonal Singh[24], MD, MPH

1. Department of Epidemiology and Biostatistics, Milken Institute School of Public Health, The George Washington University, Washington, District of Columbia
2. Center for Drug Safety and Effectiveness, Johns Hopkins Bloomberg School of Public Health, Baltimore, Maryland
3. Department of Health Policy & Management, Bloomberg School of Public Health, Baltimore,
4. Division of General Internal Medicine, Johns Hopkins Medicine, Baltimore, Maryland

Correspondence

Hsien-Yen Chang, PhD
624 N. Broadway, Room 682
Baltimore, MD, 21205
Email: hchang24@jhmi.edu
Fax: (410)-955-0470
Telephone: (410)-614-5812

Appendix 1: Subject's potential indications of dabigatran use by age groups at the start of observation (N=1215).

Age groups/ Indications	N of subjects (% among subjects of the same age group)				
	Atrial fibrillation (AF) only	Venous thromboembolism (VTE) only	AF or VTE	Other indications	Total
18-44	16 (69.57)	3 (13.04)	1 (4.35)	3 (13.04)	23
45-54	80 (77.67)	7 (6.80)	9 (8.74)	7 (6.80)	103
55-64	304 (85.15)	8 (2.24)	35 (9.80)	10 (2.80)	357
65+	576 (78.69)	10 (1.37)	120 (16.39)	26 (3.55)	732
Total	976 (80.33)	28 (2.30)	165 (13.58)	46 (3.79)	1215

Appendix 2: Administrative codes adopted to define conditions

Table 1. Administrative codes used to identify diagnosis of gastrointestinal bleeding.

ICD-9 **Code**	
531	Gastric ulcer
532	Duodenal ulcer
533	Peptic ulcer site unspecified*
534	Gastrojejunal ulcer
569.82	Ulceration of intestine
569.83	Perforation of intestine
578	Gastrointestinal hemorrhageɫ
562	Diverticula of intestine
**Current Procedural Terminology Code**	
43200, 43201, 43202, 43204, 43205, 43215, 43217, 43219, 43220, 43226, 43227, 43228, 43231, 43232, 43234, 43235, 43236, 43237, 43238, 43239, 43241, 43242, 43243, 43244, 43245, 43246, 43247, 43248, 43249, 43250, 43251, 43255, 43256, 43257, 43258, 44300, 44310, 44312, 44314, 44316, 44320, 44340, 44345, 44346, 44360, 44361, 44364, 44365, 44366, 44369, 44372, 44373, 44376, 44377, 44378, 44380, 44382, 44383, 44386, 44388, 44389, 44391, 44392, 44393, 44394	Upper gastrointestinal surgery/procedure
44389, 44391, 44392, 44393, 44394, 45300, 45305, 45307, 45308, 45309, 45315, 45317, 45320, 45330, 45331, 45333, 45334, 45335, 45355, 45378, 45379, 45380, 45381, 45382, 45383, 45384, 45385, 82270, 82271, 82272, A4773, G0394	Lower gastrointestinal surgery/procedure

* along with an upper gastrointestinal surgery or procedure within 7 days of the ICD code.

ɫ along with an upper or lower gastrointestinal surgery or procedure within 7 days of the ICD code.

ICD-9 = International Classification of Diseases, Ninth Revision.

Table 2. Administrative codes used to identify diagnosis of related co-existing conditions and indications.

***ICD-9* Code**	**Conditions**
Chronic kidney disease (585)	
585.1	Chronic kidney disease, Stage I
585.2	Chronic kidney disease, Stage II (mild)
585.3	Chronic kidney disease, Stage III (moderate)
585.4	Chronic kidney disease, Stage IV (severe)
585.5	Chronic kindey disease, Stage V
585.6	End stage renal disease
585.9	Chronic kidney disease, unspecified
Trauma (800-959)	
800-804	*Fracture of Skull*
800	Fracture of vault of skull
801	Fracture of base of skull
802	Facture of face bones
803	Other and unspecified skull fractures
804	Multiple fractures involving skull or face with other bones
805-809	*Fracture Of Spine And Trunk*
805	Fracture of vertebral column without mention of spinal cord injury
806	Fracture of vertebral column with spinal cord injury
807	Fracture of rib(s) sternum larynx and trachea
808	Fracture of pelvis
809	Ill-defined fractures of bones of trunk
810-819	*Fracture Of Upper Limb*
810	Fracture of clavicle
811	Fracture of scapula
812	Fracture of humerus
813	Fracture of radius and ulna
814	Fracture of carpal bone(s)
815	Fracture of metacarpal bone(s)
816	Fracture of one or more phalanges of hand
817	Multiple fractures of hand bones
818	Ill-defined fractures of upper limb
819	Multiple fractures involving both upper limbs and upper limb with rib(s) and sternum
820-829	*Fracture of Lower Limb*
820	Fracture of neck of femur
821	Fracture of other and unspecified parts of femur
822	Fracture of patella
823	Fracture of tibia and fibula
824	Fracture of ankle
825	Fracture of one or more tarsal and metatarsal bones
826	Fracture of one or more phalanges of foot
827	Other multiple and ill-defined fractures of lower limb
828	Multiple fractures involving both lower limbs lower with upper limb

	and lower limb(s) with rib(s) and sternum
829	Fracture of unspecified bones
830-839	*Dislocation*
830	Dislocation of jaw
831	Dislocation of shoulder
832	Dislocation of elbow
833	Dislocation of wrist
834	Dislocation of finger
835	Dislocation of hip
836	Dislocation of knee
837	Dislocation of ankle
838	Dislocation of foot
839	Other multiple and ill-defined dislocations
840-848	*Sprains And Strains Of Joints And Adjacent Muscles*
840	Sprains and strains of shoulder and upper arm
841	Sprains and strains of elbow and forearm
842	Sprains and strains of wrist and hand
843	Sprains and strains of hip and thigh
844	Sprains and strains of knee and leg
845	Sprains and strains of ankle and foot
846	Sprains and strains of sacroiliac region
847	Sprains and strains of other and unspecified parts of back
848	Other and ill-defined sprains and strains
850-854	*Intracranial Injury, Excluding Those With Skull Fracture*
850	Concussion
851	Cerebral laceration and contusion
852	Subarachnoid subdural and extradural hemorrhage following injury
853	Other and unspecified intracranial hemorrhage following injury
854	Intracranial injury of other and unspecified nature
860-869	*Internal Injury Of Chest, Abdomen, And Pelvis*
860	Traumatic pneumothorax and hemothorax
861	Injury to heart and lung
862	Injury to other and unspecified intrathoracic organs
863	Injury to gastrointestinal tract
864	Injury to liver
865	Injury to spleen
866	Injury to kidney
867	Injury to pelvic organs
868	Injury to other intra-abdominal organs
869	Internal injury to unspecified or ill-defined organs
870-879	*Open Wound Of Head, Neck, And Trunk*
870	Open wound of ocular adnexa
871	Open wound of eyeball
872	Open wound of ear
873	Other open wound of head
874	Open wound of neck

875	Open wound of chest (wall)
876	Open wound of back
877	Open wound of buttock
878	Open wound of genital organs (external) including traumatic amputation
879	Open wound of other and unspecified sites except limbs
880-887	*Open Wound Of Upper Limb*
880	Open wound of shoulder and upper arm
881	Open wound of elbow forearm and wrist
882	Open wound of hand except finger(s) alone
883	Open wound of finger(s)
884	Multiple and unspecified open wound of upper limb
885	Traumatic amputation of thumb (complete) (partial)
886	Traumatic amputation of other finger(s) (complete) (partial)
887	Traumatic amputation of arm and hand (complete) (partial)
890-897	*Open Wound Of Lower Limb*
890	Open wound of hip and thigh
891	Open wound of knee leg (except thigh) and ankle
892	Open wound of foot except toe(s) alone
893	Open wound of toe(s)
894	Multiple and unspecified open wound of lower limb
895	Traumatic amputation of toe(s) (complete) (partial)
896	Traumatic amputation of foot (complete) (partial)
897	Traumatic amputation of leg(s) (complete) (partial)
900-904	*Injury to Blood Vessels*
900	Injury to blood vessels of head and neck
901	Injury to blood vessels of thorax
902	Injury to blood vessels of abdomen and pelvis
903	Injury to blood vessels of upper extremity
904	Injury to blood vessels of lower extremity and unspecified sites
905-909	*Late Effects Of Injuries, Poisonings, Toxic Effects, And Other External Causes*
905	Late effects of musculoskeletal and connective tissue injuries
906	Late effects of injuries to skin and subcutaneous tissues
907	Late effects of injuries to the nervous system
908	Late effects of other and unspecified injuries
909	Late effects of other and unspecified external causes
910-919	*Superficial Injury*
910	Superficial injury of face neck and scalp except eye
911	Superficial injury of trunk
912	Superficial injury of shoulder and upper arm
913	Superficial injury of elbow forearm and wrist
914	Superficial injury of hand(s) except finger(s) alone
915	Superficial injury of finger(s)
916	Superficial injury of hip thigh leg and ankle
917	Superficial injury of foot and toe(s)

918	Superficial injury of eye and adnexa
919	Superficial injury of other multiple and unspecified sites
920-924	*Contusion With Intact Skin Surface*
920	Contusion of face, scalp, and neck except eye(s)
921	Contusion of eye and adnexa
922	Contusion of trunk
923	Contusion of upper limb
924	Contusion of lower limb and of other and unspecified sites
925-929	*Crushing Injury*
925	Crushing injury of face scalp and neck
926	Crushing injury of trunk
927	Crushing injury of upper limb
928	Crushing injury of lower limb
929	Crushing injury of multiple and unspecified sites
930-939	*Effects Of Foreign Body Entering Through Orifice*
930	Foreign body on external eye
931	Foreign body in ear
932	Foreign body in nose
933	Foreign body in pharynx and larynx
934	Foreign body in trachea bronchus and lung
935	Foreign body in mouth esophagus and stomach
936	Foreign body in intestine and colon
937	Foreign body in anus and rectum
938	Foreign body in digestive system, unspecified
939	Foreign body in genitourinary tract
940-949	*Burns*
940	Burn confined to eye and adnexa
941	Burn of face head and neck
942	Burn of trunk
943	Burn of upper limb except wrist and hand
944	Burn of wrist(s) and hand(s)
945	Burn of lower limb(s)
946	Burns of multiple specified sites
947	Burn of internal organs
948	Burns classified according to extent of body surface involved
950-957	*Injury To Nerves And Spinal Cord*
950	Injury to optic nerve and pathways
951	Injury to other cranial nerve(s)
952	Spinal cord injury without evidence of spinal bone injury
953	Injury to nerve roots and spinal plexus
954	Injury to other nerve(s) of trunk excluding shoulder and pelvic girdles
955	Injury to peripheral nerve(s) of shoulder girdle and upper limb
956	Injury to peripheral nerve(s) of pelvic girdle and lower limb
957	Injury to other and unspecified nerves
958-959	*Certain Traumatic Complications And Unspecified Injuries*
958	Certain early complications of trauma

959	Injury other and unspecified
H pylori infection	
041.86	Helicobacter pylori (H. pylori) infection
Atrial fibrillation	
427.31	atrial fibrillation
Venous thromboembolism (VTE)[1]	
415.0	Acute corpulmonale
415.1	Pulmonary embolism and infarction<br>- Pulmonary (artery) (vein):<br>• Apoplexy<br>• Embolism<br>• Infarction (hemorrhagic)<br>• Thrombosis
415.19	Other pulmonary embolism and infarction
416.0	Primary pulmonary hypertension<br>- Idiopathic pulmonary arteriosclerosis<br>- Pulmonary hypertension (essential) (idiopathic) (primary)
416.2	Chronic pulmonary embolism
416.8	Other chronic pulmonary heart diseases<br>- Pulmonary hypertension NOS<br>- Pulmonary hypertension, secondary
416.9	Chronic pulmonary heart disease, unspecified<br>- Chronic cardiopulmonary disease<br>- Cor pulmonale (chronic) NOS<br>Code Number Description
451.1	Phlebitis and thrombophlebitis of deep vessels of<br>lower extremities
451.11	- Phlebitis and thrombophlebitis of femoral vein (deep) (superficial)
451.19	- Phlebitis and thrombophlebitis of other<br>• Femoropopliteal vein<br>• Popliteal vein<br>• Tibial vein
451.2	Phlebitis and thrombophlebitis of lower extremities, unspecified
451.8	Phlebitis and thrombophlebitis of other sites
451.89	Phlebitis and thrombophlebitis other<br>• Axillary vein<br>• Jugular vein<br>• Subclavian vein<br>• Thrombophlebitis of breast (Mondor's disease)
451.9	Phlebitis and thrombophlebitis of unspecified site
453	Other venous embolism and thrombosis
453.1	Thrombophlebitis migrans
453.2	Embolism and thrombosis of inferior vena cava
453.4	Acute venous embolism and thrombosis of deep vessels of<br>lower extremity
453.40	Acute venous embolism and thrombosis of unspecified<br>deep vessels of lower extremity

	- Deep vein thrombosis NOS<br>- DVT NOS
453.41	Acute venous embolism and thrombosis of deep vessels of proximal lower extremity<br>- Femoral<br>- Iliac<br>- Popliteal<br>- Thigh<br>- Upper leg NOS
453.42	Acute venous embolism and thrombosis of deep vessels of distal lower extremity<br>- Calf<br>- Lower leg NOS<br>- Peroneal<br>- Tibial
453.5	Chronic venous embolism and thrombosis of deep vessels of lower extremity
453.7	Chronic venous embolism and thrombosis of other specified vessels
453.8	Acute venous embolism and thrombosis of other specified veins
453.89	Acute venous embolism and thrombosis of other specified veins
453.9	Embolism and thrombosis of unspecified site<br>- Embolism of vein<br>- Thrombosis (vein)
997.2	Peripheral vascular complications<br>- Phlebitis or thrombophlebitis during or resulting from a procedure
V12.51	Personal history of venous thrombosis and embolism

ICD-9 = International Classification of Diseases, Ninth Revision.

1. Janssen Pharmaceuticals Inc. Select ICD-9-CM Codes for VTE. Retrieved September 23, 2016, from Select ICD-9-CM Codes for VTE, http://www.janssenpharmaceuticalsinc.com/assets/Select_ICD_9_CM_Codes_for_VTE.pdf

Appendix 3: Number of Subjects Experiencing Status Change throughout Observation Period per Covariate(N=1215)

Variables	N of Status Change*	%	Included in the Adjusted Model?
Age Groups	25	2.06	NO
Sex	0	0	NO
Region	0	0	NO
HASBLED Score	1090	89.71	YES
Co-medication			
Proton pump inhibitors	442	36.38	YES
Steroid	391	32.18	YES
Pgp Inhibitors	268	22.06	YES
Nonsteroidal anti-inflammatory drugs	351	28.89	YES
Chronic conditions			
renal failure	179	14.73	NO
trauma	539	44.36	YES
H.pylori infection	22	1.81	NO

*The subject's covariate status experienced at least one change from one 3-month summary period to next(e.g. subject's HASBLED score is 0 in the first 3 months but became 2 during month 4 and the end of month 6).

Appendix 4: Incidence rate ratio and 95% confidence interval for exposures

Unadjusted Model

Variable	Level	IRR	95% CI		P-value
Benchmark analysis: 14 days of exposure grace period					
Drug	Dabigatrin vs. non-exposed	1.23	1.09	1.39	0.0007
	Warfarin vs non-exposed	1.21	0.93	1.57	0.1617
	Dabigatran vs. warfarin	1.02	0.77	1.34	0.9043
Sensitivity Analysis: 14 days of dabigatran grace period, 30 days of warfarin grace period					
Drug	Dabigatrin vs. non-exposed	1.21	1.08	1.37	0.0015
	Warfarin vs non-exposed	1.21	0.93	1.57	0.1550
	Dabigatran vs. warfarin	1.00	0.76	1.32	0.9797
Sensitivity analysis: 7 days of exposure grace period					
Drug	Dabigatrin vs. non-exposed	1.25	1.11	1.41	0.0002
	Warfarin vs non-exposed	1.26	0.97	1.65	0.0844
	Dabigatran vs. warfarin	0.99	0.75	1.31	0.9549
Sensitivity Analysis: 7 days of dabigatran grace period, 30 days of warfarin grace period					
drug	Dabigatrin vs. non-exposed	1.23	1.09	1.39	0.0009
	Warfarin vs non-exposed	1.17	0.89	1.53	0.2516
	Dabigatran vs. warfarin	1.05	0.79	1.39	0.7334

Adjusted model

Variable	Level	IRR	95% CI		P-value
Benchmark analysis: 14 days of exposure grace period					
Drug	Dabigatrin vs. non-exposed	1.01	0.90	1.15	0.8206
	Warfarin vs non-exposed	1.02	0.78	1.33	0.8803
	Dabigatran vs. warfarin	0.99	0.75	1.31	0.9644
Bleeding Score	1 vs 0	2.17	1.78	2.66	<.0001
	>=2 vs 0	4.42	3.47	5.63	<.0001
NSAID	Yes vs no	0.92	0.73	1.16	0.5011
PPI	Yes vs no	2.48	2.05	3.01	<.0001
Steroid	Yes vs no	1.14	0.92	1.41	0.2189
Pgp Inhibitor	Yes vs no	1.20	0.94	1.52	0.1351
Trauma	Yes vs no	0.98	0.82	1.18	0.8297
Sensitivity Analysis: 14 days of dabigatran grace period, 30 days of warfarin grace period					
Drug	Dabigatrin vs. non-exposed	1.00	0.88	1.13	0.9825
	Warfarin vs non-exposed	1.01	0.78	1.32	0.9247
	Dabigatran vs. warfarin	0.99	0.75	1.30	0.9193
Bleeding Score	1 vs 0	2.21	1.81	2.71	<.0001

	>=2 vs 0	4.51	3.54	5.75	<.0001
NSAID	Yes vs no	0.89	0.71	1.12	0.3210
PPI	Yes vs no	2.48	2.04	3.01	<.0001
Steroid	Yes vs no	1.14	0.93	1.41	0.2131
Pgp Inhibitor	Yes vs no	1.22	0.96	1.56	0.1041
Trauma	Yes vs no	0.97	0.80	1.16	0.7034
Sensitivity analysis: 7 days of exposure grace period					
Drug	Dabigatrin vs. non-exposed	1.04	0.92	1.17	0.5528
	Warfarin vs non-exposed	1.07	0.82	1.39	0.6223
	Dabigatran vs. warfarin	0.97	0.74	1.28	0.8344
Bleeding Score	1 vs 0	2.17	1.77	2.65	<.0001
	>=2 vs 0	4.41	3.47	5.62	<.0001
NSAID	Yes vs no	0.93	0.74	1.17	0.5526
PPI	Yes vs no	2.47	2.04	2.99	<.0001
Steroid	Yes vs no	1.13	0.91	1.39	0.2687
Pgp Inhibitor	Yes vs no	1.19	0.94	1.51	0.1526
Trauma	Yes vs no	0.97	0.81	1.16	0.7466
Sensitivity Analysis: 7 days of dabigatran grace period, 30 days of warfarin grace period					
Drug	Dabigatrin vs. non-exposed	1.02	0.90	1.15	0.8150
	Warfarin vs non-exposed	0.99	0.76	1.30	0.9531
	Dabigatran vs. warfarin	1.02	0.77	1.35	0.8713
Bleeding Score	1 vs 0	2.20	1.80	2.69	0.0437
	>=2 vs 0	4.45	3.49	5.68	<.0001
NSAID	Yes vs no	0.91	0.72	1.14	0.4033
PPI	Yes vs no	2.44	2.01	2.96	<.0001
Steroid	Yes vs no	1.13	0.92	1.40	0.2522
Pgp Inhibitor	Yes vs no	1.21	0.94	1.54	0.1360
Trauma	Yes vs no	0.98	0.81	1.17	0.7896

Appendix 5: Incidence rate ratio and 95% confidence interval for stratified analysis on HASBLED score.

All stratified analyses used 14 days of grace period for warfarin and dabigatran

Variable	Level	IRR	95% CI		P-value
Risk periods restricted to those with HASBLED=0					
Drug	Dabigatrin vs. non-exposed	1.03	0.71	1.51	0.8683
	Warfarin vs non-exposed	1.58	0.62	4.02	0.3344
	Dabigatran vs. warfarin	0.65	0.25	1.72	0.3895
NSAID	Yes vs no	0.45	0.22	0.93	0.0300
PPI	Yes vs no	1.92	1.07	3.46	0.0291
Steroid	Yes vs no	1.06	0.52	2.17	0.8745
Pgp Inhibitor	Yes vs no	1.35	0.55	3.29	0.5114
Trauma	Yes vs no	0.71	0.35	1.43	0.3343
Risk periods restricted to those with HASBLED>=1					
Drug	Dabigatrin vs. non-exposed	1.05	0.92	1.20	0.4624
	Warfarin vs non-exposed	1.05	0.79	1.40	0.7128
	Dabigatran vs. warfarin	1.00	0.74	1.33	0.9801
NSAID	Yes vs no	0.98	0.76	1.26	0.8629
PPI	Yes vs no	2.74	2.22	3.38	<.0001
Steroid	Yes vs no	1.22	0.97	1.53	0.0890
Pgp Inhibitor	Yes vs no	1.19	0.92	1.53	0.1961
Trauma	Yes vs no	1.09	0.90	1.32	0.3776

Appendix 6: Incidence rate ratio and 95% confidence interval for stratified analysis on HASBLED score.

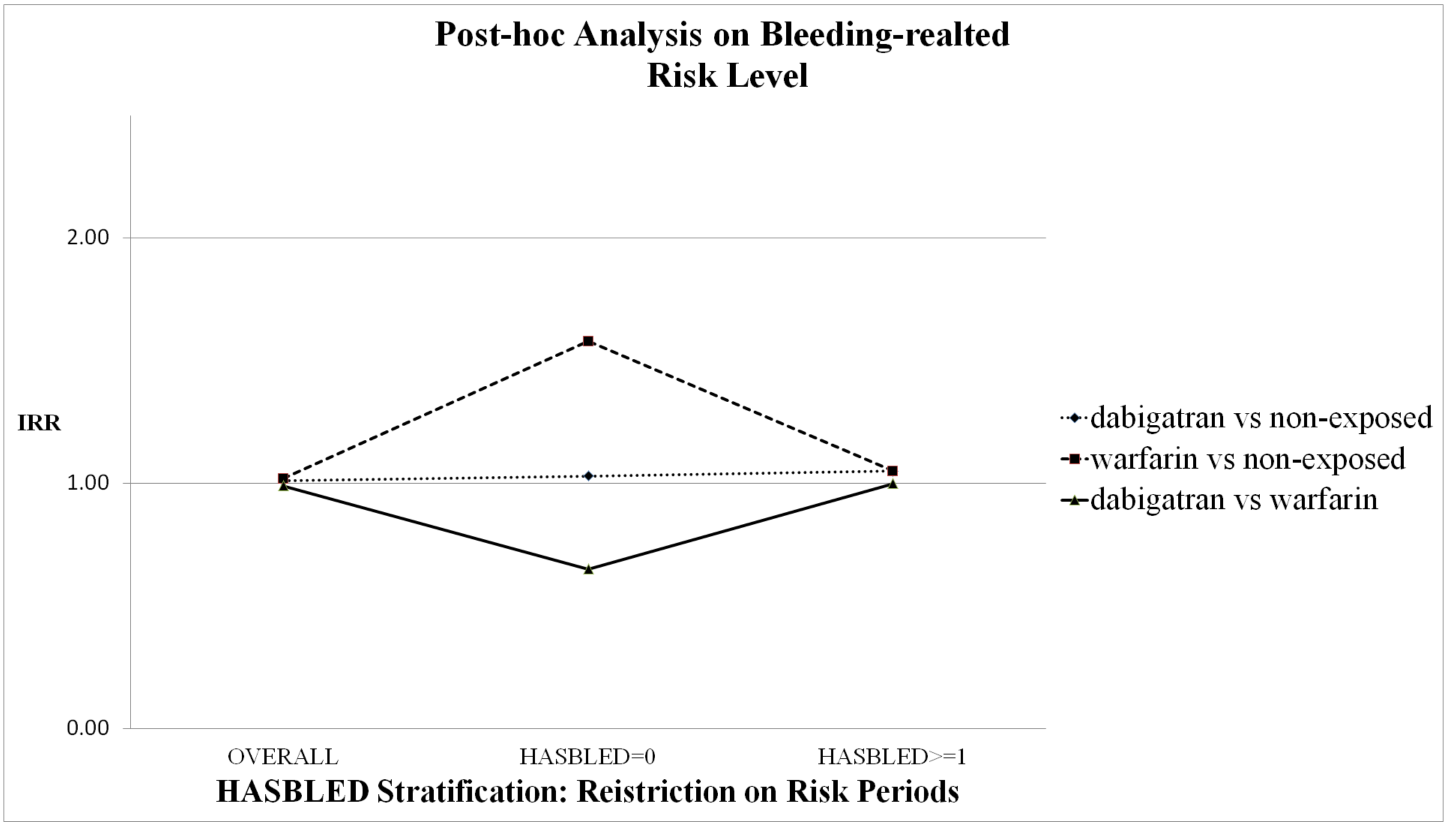

Appendix 7: Incidence rate ratio and 95% confidence interval for stratified analysis on age groups.

All stratified analyses used 14 days of grace period for warfarin and dabigatran

Variable	Level	IRR	95% CI		P-value
Risk periods restricted to those Age>65 at baseline.					
Drug	Dabigatrin vs. non-exposed	1.02	0.90	1.15	0.8074
	Warfarin vs non-exposed	0.94	0.71	1.25	0.6774
	Dabigatran vs. warfarin	1.08	0.81	1.44	0.6099
NSAID	Yes vs no	0.93	0.73	1.18	0.5357
PPI	Yes vs no	2.46	2.02	3.01	0.0000
Steroid	Yes vs no	1.18	0.95	1.47	0.1358
Pgp Inhibitor	Yes vs no	1.16	0.90	1.49	0.2416
Trauma	Yes vs no	1.01	0.84	1.22	0.9152
Bleeding Score	1 vs 0	2.39	1.90	3.00	0.0000
	>=2 vs 0	4.94	3.79	6.43	0.0000
Risk periods restricted to those Age<=65 at baseline.					
Drug	Dabigatrin vs. non-exposed	0.98	0.58	1.65	0.9435
	Warfarin vs non-exposed	2.73	1.14	6.53	0.0236
	Dabigatran vs. warfarin	0.36	0.14	0.93	0.0343
NSAID	Yes vs no	0.99	0.47	2.11	0.9835
PPI	Yes vs no	3.18	1.51	6.68	0.0023
Steroid	Yes vs no	0.81	0.36	1.84	0.6221
Pgp Inhibitor	Yes vs no	1.75	0.64	4.78	0.2734
Trauma	Yes vs no	0.58	0.25	1.32	0.1910
Bleeding Score	1 vs 0	1.52	0.95	2.43	0.0812
	>=2 vs 0	1.20	0.40	3.55	0.7472

Appendix 8: Incidence rate ratio and 95% confidence interval for exposures, including prevalent warfarin users in study population

Unadjusted Model

Variable	Level	IRR	95% CI		P-value
Benchmark analysis: 14 days of exposure grace period					
Drug	Dabigatrin vs. non-exposed	1.29	1.17	1.42	<.0001
	Warfarin vs non-exposed	1.11	0.98	1.26	0.0857
	Dabigatran vs. warfarin	1.16	1.03	1.30	0.0153

Adjusted model

Variable	Level	IRR	95% CI		P-value
Benchmark analysis: 14 days of exposure grace period					
Drug	Dabigatrin vs. non-exposed	1.12	1.02	1.23	0.0186
	Warfarin vs non-exposed	1.00	0.88	1.13	0.9638
	Dabigatran vs. warfarin	1.13	1.00	1.27	0.0528
Bleeding Score	1 vs 0	1.94	1.65	2.28	<.0001
	>=2 vs 0	3.91	3.23	4.72	<.0001
NSAID	Yes vs no	0.97	0.81	1.17	0.7565
PPI	Yes vs no	2.63	2.28	3.04	<.0001
Steroid	Yes vs no	1.15	0.99	1.35	0.0751
Pgp Inhibitor	Yes vs no	0.93	0.77	1.14	0.4926
Trauma	Yes vs no	1.04	0.91	1.18	0.5836

Appendix 9: STROBE Statement—Checklist of items that should be included in reports of cohort studies

	Item No	Recommendation	Page #
**Title and abstract**	1	(a) Indicate the study's design with a commonly used term in the title or the abstract	1
		(b) Provide in the abstract an informative and balanced summary of what was done and what was found	2
**Introduction**			
Background/rationale	2	Explain the scientific background and rationale for the investigation being reported	3
Objectives	3	State specific objectives, including any prespecified hypotheses	4
**Methods**			
Study design	4	Present key elements of study design early in the paper	5 & Figure 1
Setting	5	Describe the setting, locations, and relevant dates, including periods of recruitment, exposure, follow-up, and data collection	6&7
Participants	6	(a) Give the eligibility criteria, and the sources and methods of selection of participants. Describe methods of follow-up	5&6
		(b) For matched studies, give matching criteria and number of exposed and unexposed	
Variables	7	Clearly define all outcomes, exposures, predictors, potential confounders, and effect modifiers. Give diagnostic criteria, if applicable	6&7 & Appendix 1-3
Data sources/ measurement	8*	For each variable of interest, give sources of data and details of methods of assessment (measurement). Describe comparability of assessment methods if there is more than one group	6&7
Bias	9	Describe any efforts to address potential sources of bias	7&8
Study size	10	Explain how the study size was arrived at	6
Quantitative variables	11	Explain how quantitative variables were handled in the analyses. If applicable, describe which groupings were chosen and why	6-8
Statistical methods	12	(a) Describe all statistical methods, including those used to control for confounding	7&8
		(b) Describe any methods used to examine subgroups and interactions	8
		(c) Explain how missing data were addressed	NA
		(d) If applicable, explain how loss to follow-up was addressed	NA
		(e) Describe any sensitivity analyses	8
**Results**			
Participants	13*	(a) Report numbers of individuals at each stage of study—eg numbers potentially eligible, examined for eligibility, confirmed eligible, included in the study, completing follow-up, and analysed	9
		(b) Give reasons for non-participation at each stage	NA
		(c) Consider use of a flow diagram	NA
Descriptive data	14*	(a) Give characteristics of study participants (eg demographic, clinical, social) and information on exposures and potential confounders	9 & Table 1, Appendix 1&3
		(b) Indicate number of participants with missing data for each variable of interest	9 & Table 1
		(c) Summarise follow-up time (eg, average and total amount)	11 & Table 2
Outcome data	15*	Report numbers of outcome events or summary measures over time	11 & Table 2

Main results	16	(a) Give unadjusted estimates and, if applicable, confounder-adjusted estimates and their precision (eg, 95% confidence interval). Make clear which confounders were adjusted for and why they were included	11&12 & Table 3
		(b) Report category boundaries when continuous variables were categorized	11&12& Table 3, Appendix 3
		(c) If relevant, consider translating estimates of relative risk into absolute risk for a meaningful time period	NA
Other analyses	17	Report other analyses done—eg analyses of subgroups and interactions, and sensitivity analyses	12&13 & Appendix 4-8
**Discussion**			
Key results	18	Summarise key results with reference to study objectives	13
Limitations	19	Discuss limitations of the study, taking into account sources of potential bias or imprecision. Discuss both direction and magnitude of any potential bias	15&16
Interpretation	20	Give a cautious overall interpretation of results considering objectives, limitations, multiplicity of analyses, results from similar studies, and other relevant evidence	13-15
Generalisability	21	Discuss the generalisability (external validity) of the study results	14
**Other information**			
Funding	22	Give the source of funding and the role of the funders for the present study and, if applicable, for the original study on which the present article is based	NA

*Give information separately for exposed and unexposed groups.

Note: An Explanation and Elaboration article discusses each checklist item and gives methodological background and published examples of transparent reporting. The STROBE checklist is best used in conjunction with this article (freely available on the Web sites of PLoS Medicine at http://www.plosmedicine.org/, Annals of Internal Medicine at http://www.annals.org/, and Epidemiology at http://www.epidem.com/). Information on the STROBE Initiative is available at http://www.strobe-statement.org.
